# Performance comparison of two whole genome amplification techniques in frame of multifactor preimplantation genetic testing

**DOI:** 10.1007/s10815-018-1187-4

**Published:** 2018-04-23

**Authors:** Ludmila Volozonoka, Dmitry Perminov, Liene Korņejeva, Baiba Alkšere, Natālija Novikova, Evija Jokste Pīmane, Arita Blumberga, Inga Kempa, Anna Miskova, Linda Gailīte, Violeta Fodina

**Affiliations:** 1 Scientific Laboratory of Molecular Genetics, Riga Stradins University, Dzirciema street 16, Riga, LV-1007 Latvia; 2 Centre of Genetics, “IVF Riga” Reproductive Genetics Clinic, Riga, LV-1010 Latvia; 3 Department of Molecular Biology, “E. Gulbja Laboratory”, Riga, LV-1006 Latvia; 4 Faculty of Medicine, University of Latvia, Riga, LV-1586 Latvia; 5 Department of Obstetrics and Gynecology, Riga Stradins University, Riga, LV-1007 Latvia

**Keywords:** Embryo, Preimplantation genetic testing, Single gene disorder, Aneuploidy, Whole genome amplification

## Abstract

**Purpose:**

To compare multiple displacement amplification and OmniPlex whole genome amplification technique performance during array comparative genome hybridization (aCGH), Sanger sequencing, SNaPshot and fragment size analysis downstream applications in frame of multifactor embryo preimplantation genetic testing.

**Methods:**

Preclinical workup included linked short tandem repeat (STR) marker selection and primer design for loci of interest. It was followed by a family haplotyping, after which an in vitro fertilization preimplantation genetic testing (IVF-PGT) cycle was carried out. A total of 62 embryos were retrieved from nine couples with a confirmed single gene disorder being transmitted in their family with various inheritance traits—autosomal dominant (genes—*ACTA2*, *HTT*, *KRT14*), autosomal recessive (genes—*ALOX12B*, *TPP1*, *GLB1*) and X-linked (genes—*MTM1*, *DMD*). Whole genome amplification (WGA) for the day 5 embryo trophectoderm single biopsies was carried out by multiple displacement amplification (MDA) or polymerase chain reaction (PCR)-based technology OmniPlex and was used for direct (Sanger sequencing, fragment size analysis, SNaPshot) and indirect mutation assessment (STR marker haplotyping), and embryo aneuploidy testing by array comparative genome hybridization (aCGH).

**Results:**

Family haplotyping revealed informative/semi-informative microsatellite markers for all clinical cases for all types of inheritance. Indirect testing gave a persuasive conclusion for all embryos assessed, which was confirmed through direct testing. The overall allele dropout (ADO) rate was higher for PCR-based WGA, and MDA shows a better genomic recovery scale. Five euploid embryos were subjected to elective single embryo transfer (eSET), which resulted in four clinical pregnancies and birth of two healthy children, which proved free of disease causative variants running in the family postnataly.

**Conclusions:**

A developed multifactor PGT protocol can be adapted and applied to virtually any genetic condition and is capable of improving single gene disorder preimplantation genetic testing in a patient-tailored manner thus increasing pregnancy rates, saving costs and increasing patient reliability.

**Electronic supplementary material:**

The online version of this article (10.1007/s10815-018-1187-4) contains supplementary material, which is available to authorized users.

## Introduction

Preimplantation genetic testing (PGT), formerly known as PGD for monogenic disease testing or PGS for chromosome screening, is an alternative to prenatal testing for couples being at risk of transmitting a genetic disorder to their offspring [38]. PGT allows exclusion of affected embryos before a clinical pregnancy has been established thus avoiding invasive prenatal testing and elective termination of pregnancy due to prenatally confirmed diagnosis. The material for PGT can be collected from day 3 or day 5 of developing embryo before its transfer to the uterus. The process initially requires controlled ovarian hyperstimulation, oocyte retrieval and subsequent oocyte in vitro fertilization (IVF), most commonly by intracytoplasmic sperm injection (ICSI) followed by embryo cultivation until the desired stage of development as well as a biopsy procedure [1]. Depending on a protocol, PGT can be done with or without embryo vitrification for the time of testing. Only embryos proved free of the disease-causing variant under consideration are subsequently transferred into the uterine cavity.

The success of the whole procedure depends mostly on competence and appropriate collaboration of the multidisciplinary team consisting of a clinical geneticist, reproductologist, gynaecologist, embryologist and molecular geneticist, and is achieved through safety and accuracy, improving genetic and reproductive medicine practices [6]. PGT is currently performed for single gene disorders (SGDs), late-onset disorders with genetic predisposition, chromosomal disorders, including aneuploidy and structural rearrangements, and HLA (human leukocyte antigen) typing to improve the access to HLA-matched stem cell transplantation [28].

The history of PGT goes back to 1989 when A. Handyside performed first preimplantation genetic diagnostic (PGD) cases detecting a Y chromosome-specific region with PCR in case of X-linked adrenoleukodystrophy and X-linked mental retardation [13]. Now, defining embryo gender is known as sexing and can complement to genetic testing of monogenic disorders linked to the sex chromosomes.

With time, PGT underwent significant methodological and approach changes, starting from polar body testing and blastomere analysis and now adapting trophectoderm biopsy with subsequent blastocyst freezing [26]. The analysis of more than a single cell leads to a more robust downstream molecular investigation, which sets among the reasons blastocyst stage biopsy strategy [5]. Molecular genetic testing developed from single loci directs PCR till sophisticated single cell whole genome amplification [9]. Embryo haplotyping offers a more generic approach to preimplantation diagnosis, and is especially useful for diseases with a wide spectrum of causative variants, such as cystic fibrosis and Duchenne muscular dystrophy [26].

Despite technological improvements, development of PGT protocols is challenging and prone to amplification failure, DNA contamination and ADO (allele dropout)—a phenomenon common to all single cell-based PCR tests, thus affecting the reliability of the test. ADO can be defined as amplification failure affecting only one of the parental alleles. ADO’s incidence varies, but in extreme cases has affected 20% of amplifications and in the past has led to several misdiagnoses [3]. The causes of misdiagnosis include swap of samples, transfer of the wrong embryo, maternal or paternal contamination, ADO, use of inappropriate probes or primers, probe or primer failure and chromosomal mosaicism [15].

ADO rates should be as low as possible, preferably less than 10%. Higher ADO rates can be tolerated when dealing with WGA-based protocols and autosomal recessive diseases compared to autosomal dominant or compound heterozygous cases. However, in such cases an increased number of linked markers have to be used [15].

Choosing the WGA type is also challenging due to difficulties in interpretation of downstream applications like short tandem repeat (STR) marker sizing with fluorescent polymerase chain reaction (fPCR) or array comparative genomic hybridization [25]. At the moment, several WGA technologies exist [40], for example, PCR-based approaches like degenerate oligonucleotide primer (DOP) [31] or primer extension (PEP) PCR technology [39]. Leading positions are taken by OmniPlex linear WGA [4, 33] technology developed by Rubicon Genomics and multiple displacement isothermal synthesis by a Phi29 polymerase approach (Alan H. [14]). Both of them have advantages and disadvantages. The use of *Taq* DNA polymerase in PCR-based approaches limits the fragment lengths to 3 kb. The Phi29 polymerase used for MDA generates DNA fragments up to 100 kb and has a 3′ → 5′ exonuclease proofreading activity. Often, it is not clear which technology could be prioritized in custom-designed protocols [40].

Better PGT results are now achieved through combining direct and indirect testing, using platforms like Karyomapping [12] for genome-wide linkage analysis or turning to next generation sequencing (NGS) protocols (Francesco [10]). However, current studies still highlight clinically important limitations in the reliability of the technologies; for example, using Karyomapping, ∼ 14% of embryos are expected to remain without a conclusive result [19]. NGS has potential power to increase throughput and evaluate multiple genetic loci in parallel, but it is also well known for sequencing artefacts, which may complicate its application to PGD [32]. Also, costs are still quite high especially for limited sample amounts.

Regardless of the fact that PGT is recognized for its benefits, it is still relatively unregulated and lacks standardization compared with other forms of diagnostic testing [15]. This is partially because PGD lies at the intersection of two technologies with a confusing regulatory status: assisted reproduction and genetic testing [8]. It is admitted that a robust PGT test should be able not only to distinguish between a normal and affected embryo, but also to highlight all the unexpected events that may happen during meiosis, fertilization or PGD experimental procedure, and thus it should detect recombination, monosomy or trisomy and therefore diagnose abnormal embryos and detect ADO and contamination [18]. In case of adverse misdiagnosis, lessons can be very painful to patients and staff [16, 36].

Despite numerous advances, assisted reproductive technology (ART) live birth rates are still low ranging from 27 to 55%, depending on the patient age group and methodology used [6]. Another step in reaching considerably good results for SGD-PGT is embryo aneuploidy exclusion since it is well known that preimplantation human embryos are prone to chromosome instability [34] and high aneuploidy rates [18, 35]. Early results show that combined PGD and PGS increase patient chance for a healthy childbirth [20, 30].

Taking into account the aforementioned information, the aim of our study was to develop an individualized effective and robust multifactor embryo testing protocol and show a performance comparison of two WGA techniques in four different downstream applications—STR sizing, Sanger sequencing, aCGH and SNaPshot technology. We present our PGT experience for single gene diseases of autosomal dominant (genes: *ACTA2*, *HTT*, *KRT14*), autosomal recessive (genes: *ALOX12B*, *TPP1*, *GLB1*) and X-linked (genes: *MTM1*, *DMD*) types of inheritance.

## Materials and methods

### Cases processed

Nine couples (Table 1) with confirmed particular single gene disease being transmitted in their family underwent counselling regarding PGT procedure, ovarian stimulation, oocyte aspiration and IVF at the single centre of infertility and reproductive genetics where all biological material samples were collected and processed.

**Table 1:** Processed PGT cases description

PGD case	Disease	Gene	Type of inheritance	Mutation(s) assessed	Female partner age	Male partner age	Family members analyzed to establish linkage
HTT-case	Huntington disease (HD)	*HTT*	AD	CAG repeat expansion	34	29	Carrier female partner, her affected father and healthy mother, healthy male partner
ACTA2-case	Familial thoracic aortic aneurysm and dissection	*ACTA2*	AD	c.635G>A	32	32	Affected male partner, his affected mother and brother, healthy father, healthy female partner
KRT14-case	Epidermolysis bullosa simplex	*KRT14*	AD	c.374G>A	30	31	Affected male partner, healthy female partner, their affected child
TPP1-case	Classic late infantile neuronal ceroid lipofuscinosis	*TPP1*	AR	c.622C>T	38	37	Carrier female and male partners, their affected child
ALOX12B-case	Nonbullous congenital ichthyosiform erythroderma (NBCIE)	*ALOX12B*	AR	c.883G>C; c.1790C>A	35	32	Carrier female and male partners, their affected child
DMD-case 1	Duchenne muscular dystrophy (family 1)	*DMD*	X-linked	Duplication of exons 45–47 and 51–52	39	35	Carrier female partner, healthy male partner, their affected son and carrier daughter
DMD-case 2	Duchenne muscular dystrophy (family 2)	*DMD*	X-linked	c.6420del	33	31	Carrier female partner, her affected and healthy brothers, healthy male partner
MTM1-case	Myotubular myopathy	*MTM1*	X-linked	c.70C>T	34	35	Carrier female partner, her healthy sister, her carrier mother and healthy father, healthy male partner, their affected child
GLB1-case	GM1 gangliosidosis	*GLB1*	AR	c.1768C>T; c.833delG	36	38	Carrier female and male partners, their affected child
				Average	34.4 ± 2.8	32.8 ± 2.5	

### Compliance with ethical standards

The study is in accordance with the Declaration of Helsinki ethical principles. All patients considered for PGT underwent genetic counselling. Procedures and manipulations needed for embryo genetic testing were explained in detail, and signed informed consent was obtained. The study protocol was approved by a local ethical community. PGD is recognized as “an established procedure with specific and expanding applications for standard clinical practice” by the Practice Committee of the American Society for Reproductive Medicine and the Practice Committee of the Society for Assisted Reproductive Technology (2006). No research was conducted on the embryos. All genetic conditions for which PGT was performed are approved by HFEA (Human Fertilization and Embryology Act) as suitable for genetic testing in preimplantation embryos.

### Preclinical workup

Before processing a clinical case, a workup was carried out to prepare each PGT case. Linked microsatellites adjacent to the gene of interest (within ~ 2 Mb upstream and downstream from the mutation locus) were located through a University of California Santa Cruz (UCSC) genome browser (https://genome-preview.ucsc.edu/index.html). For all loci (disease-causative variant site and STR markers), semi-nested primers for two round multiplex fPCRs (inner primer fluorescently tagged with 6-FAM or HEX fluorophores at the 5′ end) were designed using “Primer-BLAST” to ensure specificity [37] following good practice guidelines [16]. Primer dimer and primer-amplicon secondary structure formation was checked using an OligoAnalyzer 3.1 online software tool [23].

DNA obtained from the peripheral venous blood of couples seeking PGD and other family members (usually three to five individuals) was isolated using a standard procedure (Qiagen). Family haplotypes flanking locus of interest were assessed. STR marker informativeness was evaluated as follows: fully informative (three or four different paternal and maternal alleles, depending on the type of inheritance, both disease-causative and healthy, are distinguishable), semi-informative (one or two different alleles can be distinguished and assigned to the normal or disease-causative haplotype) and not informative (origin of the allele or the assignment to the haplotype cannot be distinguished) [15].

When PCR linkage analysis was performed for a family, 6–13 (8.1 ± 2.5) informative or semi-informative STR markers (Table 3) were included in the following PGT cycle for embryo analysis. The STR marker informativeness rate was 53%. For autosomal recessive disorders, significantly higher STR amounts contribute to an overall assay informativeness rate: 13/15 and 10/15 compared to autosomal dominant or X-linked conditions i.e. 7/17 and 6/13 informative markers (please refer to Table 3). Disease-causative variant confirmation in family members was carried out via Sanger sequencing for single nucleotide variation (SNV) or by fragment size analysis for trinucleotide repeat expansion.

### IVF and embryo biopsy

Oocyte cumulus complexes (COC) were retrieved by a needle transvaginal aspiration procedure. All oocytes were fertilized through intracytoplasmic sperm injection (ICSI) and placed in a time-lapse incubator (EmbryoScope, Vitrolife, UK). Fertilization was acknowledged as successful if two pronuclei (PN) were observed on the next day after ICSI. Embryos were incubated until the day-5 blastocyst stage. The embryo development rate was scored based on a time-lapse system monitoring algorithm [22]. Through natural selection, the average 5th-day survival rate was 70%. In total, 62 patient embryos were subjected to PGT (Table 2). Embryo biopsies were made using a laser-assisted micromanipulator (Narishige, Japan). From each embryo, one to eight trophectodermal cells were taken from the outer layer of the blastocyst, and all embryos survived the biopsy procedure. Biopsied cells were washed in 1× phosphate-buffered saline (PBS) buffer (Cell Signaling Technologies, USA) drops to reduce the risk of contamination and subsequently placed in 0.2-ml tubes within 2.0 μl of 1% polyvinylpyrrolidone (FertiPro, Belgium) 1× PBS buffer and frozen immediately, and each blastocyst culture media contamination control was collected as well. Biopsied embryos were vitrified.

**Table 2:** Embryological data of processed cases

Case	COC retrieved	Performed ICSI	Oocytes fertilized (day 1 assessment)	Successfully fertilized oocytes^a^	Fertilization rate^b^	Embryos biopsied	Blastocyst formation rate^c^
HTT-case	16 COC (3 GV, 1 MI, 12 MII)	13	1 deg, 2 × 0 PN, 10 × 2 PN	10	0.77	7 × 2 PN	0.70
ACTA2-case	18 (1 empty ZP, 1 MI, 16 MII)	17	4 deg, 2 × 0 PN, 11 × 2 PN	11	0.65	9 × 2 PN	0.82
TPP1-case	8 COC (2 GV, 6 MII)	6	1 deg, 1 × 1 PN, 1 × 3 PN, 3 × 2 PN	4	0.67	3 × 2 PN	0.75
ALOX12B-case (two stimulations)	15 COC (1 empty ZP, 2 atretic, 3 GV, 1 MI, 8 MII)	9	2 deg, 1 × 0 PN, 2 × 3 PN, 3 × 2 PN	5	0.56	2 × 2 PN, 1 × 3 PN	0.6
	16 COC (2 empty ZP, 2 GV, 2 MI, 10 MII)	12	2 deg, 1 × 1 PN, 9 × 2 PN	9	0.75	9 × 2 PN	1.0
DMD-case 1	18 COC (1 empty ZP, 1 MI, 16 MII)	17	4 deg, 2 × 0 PN, 11 × 2 PN	11	0.65	9 × 2 PN	0.82
DMD-case 2	22 COC (2 empty ZP, 2 GV, 18 MII)	18	1 × 1 PN, 17 × 2 PN	17	0.94	16 × 2 PN, 1 × 1 PN	1.00
MTM1-case (two stimulations)	5 COC (1 GV, 4 MII)	4	3 × 0 PN, 1 × 1 PN	0	0.00	NA	NA
	10 COC (2 ZP, 1 MI, 7 MII)	8	2 deg, 4 × 0 PN, 2 × 3 PN	2	0.25	0	0.00
GLB1-case	15 COC (1 ZP, 14 MII)	14	2 × 0 PN, 2 × 1 PN, 1 × 3 PN, 9 × 2 PN	10	0.71	5 × 2 PN	0.5
	Average	11.6 ± 5.1		7.67 ± 5.34	0.58 ± 0.29	7.13 ± 5.25	0.71 ± 0.32
					Total	57.0	

*COC* cumulus oocyte complex, *ZP* zona pellucida, *GV* germinal vesicle stage oocyte, *MI* meiosis I stage oocyte, *MII* meiosis II stage oocyte, *deg* degraded oocyte

^a^Successfully fertilized oocytes are the ones having two or three pronuclei

^b^Fertilization rate is calculated dividing day 1 embryos showing two or three pronuclei (PN) with total amount of ICSI performed

^c^Blastocyst formation rate is calculated dividing day 5 embryos by successfully fertilized embryos

### Performance of clinical cases

As a first step for all embryo biopsies, WGA was carried out. For one part of the embryos (*n* = 39), WGA was done by MDA technology (SureMDA, Illumina, USA); the rest (*n* = 34) were carried out by OmniPlex linear WGA technology (SurePlex, Illumina, USA) (Table 4). Aliquots of the WGA product from each sample were used to carry out different downstream tests.

Embryo haplogroup analysis was carried out assessing informative markers found in a linkage step. Two round (nested) PCR conditions were used: 8.2 μl of Type-it Master Mix (Qiagen, USA), 0.32 μl of 0.2 μM forward (outer-forward primer for the first stage of hemi-nested PCR and inner-forward primer for the second-round PCR stage; synthesized by Bioneer, China) and 0.2 μM reverse primers (similar for both PCR steps), 6.8 μl of ddH_2_O and 0.62 μl of WGA product. Cycling conditions are as follows: initial denaturation for 5 min at 95 °C, followed by 28 cycles (first-round PCR) or 22 cycles (second-round PCR) for 30 s at 95 °C, 1 min and 30 s at 60 °C and 30 s at 72 °C and a final extension for 10 min at 72 °C. Amplified products were run on agarose gel electrophoresis to detect the PCR product. Amplicon detection was performed by capillary electrophoresis (ABI Prism 3500 DNA Analyzer; Applied Biosystems, USA). Allele sizing was carried out using GeneMapper v.4.0 software (Applied Biosystems).

Direct mutation analysis for SNVs was carried out by a standard Sanger sequencing protocol [27] or SNaPshot technology (Applied Biosystems, USA). *HTT* gene (OMIM# 613004) CAG repeat expansion (RCV000030659, HGVS nomenclature—NM_002111.6(HTT):c.53_55[(41_?)] (p.Gln40(41_?)) was detected by capillary electrophoresis using the same protocol as for STR marker loci amplification.

Embryo chromosome analysis was performed following the manufacturer’s (24sure, Illumina, USA) protocol for aCGH, shortly: the WGA product was fluorescently labelled by a nick translation method with Cy3 and Cy5 fluorophores, the reference DNA was hybridized on BAC array microchips, and microchip glasses were washed and scanned with an InnoScan (Innopsys, France) scanner. Tiff images were imported into BlueFuse Multi V4.0 software (standard settings), and the resulting copy number karyotypes were assessed. The given methodology detects unbalanced chromosomal material changes and polyploidy if sex chromosomes are represented by at least one X and Y chromosome.

ADO rates were calculated by dividing homozygous genotype events when a heterozygous genotype was expected for the number of all expected heterozygous loci. In order to access whether ADO rates are affected by downstream application, STR ADO calculations were assessed per individual case as well as per each WGA type. For Sanger sequencing and SNaPshot technologies, ADO rates we assessed separately for each technology (please refer to Table 4). ADO was counted if an alternative allele was completely absent or was hardly distinguishable from the artefact (partial ADO).

## Results

### Embryo PGT analysis

**Fig. 1:**
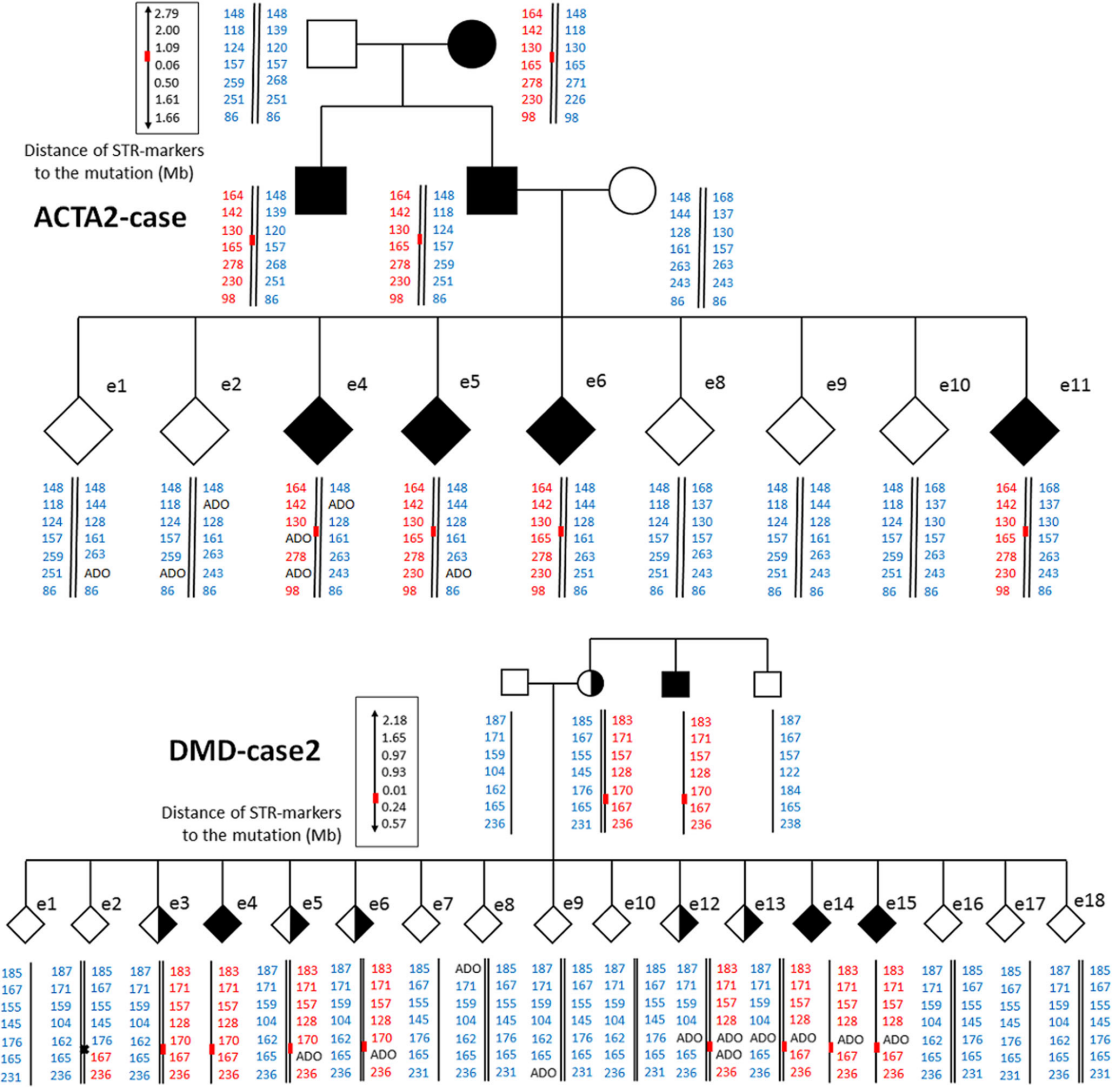
Pedigrees. DMD-case 2 and ACTA2-case pedigrees showing family members participating in haplotype establishment and all embryos analysed. Red bars represent disease variant loci. Black crosses indicate recombination events. In blue, variant-free haplotypes are indicated. In red, variant haplotypes are indicated. ADO—allelic dropout

For all 62 patient embryo biopsies, WGA amplification performed either by SureMDA or SurePlex kit (Table 4) was successful and eventually had a conclusive result (Table 3, Fig. 1 and Supplementary Figs. 1–7 for the pedigrees). Additional 11 OmniPlex samples were donated for research. In case of *MTM1* gene testing after two stimulation cycles, none of the oocytes underwent successful fertilization. The KRT14-case family underwent only linkage analysis and now are preparing for follicular stimulation.

**Table 3:** PGD results

Case	STS markers tested/informative markers	STR informativeness rate	Embryos analyzed	Affected embryo amount	Carrier embryo amount	Mutation free embryo amount	Direct mutation testing	aCGH performed	aCHG result	Overall PGT result
HTT-case	13/6	0.46	7	1; 0.14	NA	6; 0.85	yes	Performed for 2 embryos, scored highest according to ES algorithm	1 aneuploid, 1 euploid	eSET > healthy noncarrier baby born (postnatal confirmatory analysis)
ACTA2-case	18/7	0.39	9	4; 0.44	NA	5; 0.55	yes	Performed for all mutation-free embryos	4 euploid, 1 aneuploid	eSET > clinical pregnancy
TPP1-case	15/10	0.67	3	1; 0.33	1; 0.33	1; 0.33	yes	Performed for only one mutation-free embryo	Euploid	eSET > clinical pregnancy
ALOX12B-case	15/13	0.87	12	3; 0.25	7; 0.58	2; 0.17	yes	Performed for one embryo, scored highest according to the ES algorithm	Euploid	eSET > healthy noncarrier baby born (postnatal confirmatory analysis)
DMD-case 1	21/11	0.52	9	4; 1.5	1; 0.11	4; 0.44	no	PGD by aCGH performed initially when molecular analysis was unavailable; later all stored WGAs were subjected to haplotype analysis	5 euploid, 4 aneuploid	eSET > healthy carrier baby born (postnatal indirect linkage confirmatory analysis), second eSET > failed implantation
DMD-case 2	16/7	0.44	17	3; 0.18	5; 0.29	9; 0.52	yes	Performed for 9 mutation-free embryos	6 euploid, 3 aneuploid	Waiting for eSET
GLB1-case	15/8	0.53	5	0; 0	4; 0.8	1; 0.2	yes	Performed for all 5 embryos	2 euploid, 3 aneuploid	Waiting for eSET (carrier embryo)
MTM1-case	14/7	0.50	No oocytes were successfully fertilized					Pregnancy rate		0.83
KRT14-case	17/7	0.41	Ovary stimulation still to be performed							
	Average	0.53	9.5							

*ES* EmbryoScope time-lapse incubator, *eSET* elective single embryo transfer

The portion of each embryo WGA product was used for haplotyping informative or semi-informative markers detected by initial family linkage analysis. The overall STR ADO rate was 4.74% (Table 4) exceeding the 5% cut-off only in DMD-case 1, where the WGA product generated by OmniPlex had lower quality due to long-time storage and repeated freeze-thaw events. aCGH for this DMD-case 1 was performed firstly when haplotyping was unavailable (please refer to Table 3 and corresponding pedigree).

**Table 4:** WGA technique comparison

	Sanger sequencing	SNaPshot analysis	STR analysis	STR ADO (%)	aCGH analysis	Total embryos analyzed
HTT-case	–	–	7 MDA	4.5	2 MDA	7 MDA
ACTA2-case	4 MDA; 5 OmniPlex	–	4 MDA; 5 OmniPlex	1.5	3 MDA; 2 OmniPlex	4 MDA; 5 OmniPlex
TPP1-case	3 MDA	–	3 MDA	1.4	1 MDA	3 MDA
ALOX12B-case	12 MDA	12 MDA	12 MDA	2.8	2 MDA	12 MDA
DMD-case 1	–	–	9 OmniPlex	13.3	9 OmniPlex	9 OmniPlex
DMD-case 2	8 MDA; 9 OmniPlex	8 MDA; 9 OmniPlex	8 MDA; 9 OmniPlex	4.7	3 MDA; 5 OmniPlex	8 MDA; 9 OmniPlex
GLB1-case	5 MDA	–	5 MDA	2.0	5 MDA	5 MDA
WGA material donated for research	–	11 OmniPlex	–	–	11 OmniPlex	11 OmniPlex
MDA	*n* = 32, ADO 10.0%	*n* = 20, ADO 5.5%	*n* = 39, clear electropherograms	2.98	*n* = 17, noisy profiles, resolution: full chromosomes, ~ 30% of samples have to be reanalyzed	*n* = 39
OmniPlex	*n* = 14, ADO 21.4%	*n* = 20, failed reaction or inconsistent result for > 60% of cases	*n* = 23, electropherograms overrepresented with stutter peaks	6.5	*n* = 27, clear profiles, resolution: ~ 5 Mb	*n* = 34
Total	46	40	62	Average 4.74	44	73

All ADO rates were calculated by dividing homozygous genotypes when heterozygous (Hz) was expected to all expected Hz loci. ADO was counted if an alternative allele was completely absent or was indistinguishable from the artefact (partial ADO)

*ADO* allelic dropout

In one case, maternal uniparental disomy of the tested locus was observed (GLB1-case e4; please see the pedigree). In three embryos, crossover events were detected through haplotyping. TPP1-case e5 analysis was encumbered due to close proximity of the crossover site to the mutation locus making it impossible to exclude direct testing ADO and possible heterozygous embryo genotypes. In all cases, crossover occurred next to the mutation locus, which complicates particular embryo analysis, but direct mutation analysis complemented and clarified haplotyping results.

Direct mutation testing was done for all cases processed except for DMD-case 1. For *HTT* gene’s CAG, triplet repeat sizing was performed by fPCR. Sanger sequencing and/or SNaPshot analysis was applied for SNV analysis, and no mismatches were identified between direct and indirect tests. In all cases, at least one embryo free of tested disease-causative variant was detected.

In most cases, a portion of the WGA product from mutation-free embryos was subjected to aCGH analysis to exclude chromosomal aneuploidies. For the HTT-case and ALOX12B-case, only some of the tested disease-causative variant-free embryos were subjected to chromosome analysis due to financial reasons, in these cases only embryos, which showed best development scores according to the EmbryoScope algorithm, which were taken for analysis. In all cases, at least one euploid embryo was available (Table 3). Only embryos free of disease-causative variants were assessed, and euploids were rated as transferable. All embryos subjected to the embryo transfer procedure underwent thawing successfully.

Elective single euploid embryo transfers (eSET) in two cases resulted in healthy newborn babies. Transfer of *TPP1* and *ACTA2* variant-free embryos resulted in progressing clinical pregnancies. For the first Duchene muscular dystrophy case initially, only sexing for PGD by aCGH was performed and 46,XX embryo transfer resulted in a healthy carrier baby birth; only later were all their embryos haplotyped, and a second eSET resulted in failed embryo implantation. Another DMD family prepared for the eSET procedure. Three babies born after PGT underwent postnatal mutation assessment, and preimplantation genetic testing results were confirmed. The overall pregnancy rate is 83%.

### Comparison of two different WGA techniques

To compare two WGA methods, one part of the biopsies was subjected to MDA technique and the rest were amplified by an OmniPlex reagent kit (please refer to Table 4 for the detailed view). A typical MDA product pattern (smear) on 1.5% agarose gel is observable as bands at about 6–12 kb. On the contrary, PCR-based WGA results in much shorter products visible as a smear appearing between 1 kb and 100 bp with most prominent bands at around 500 pb (Supplementary Fig. 9). Both types of WGA were subjected to all four downstream applications—Sanger sequencing, STR amplification, SNaPshot and aCGH (Table 4).

**Fig. 2:**
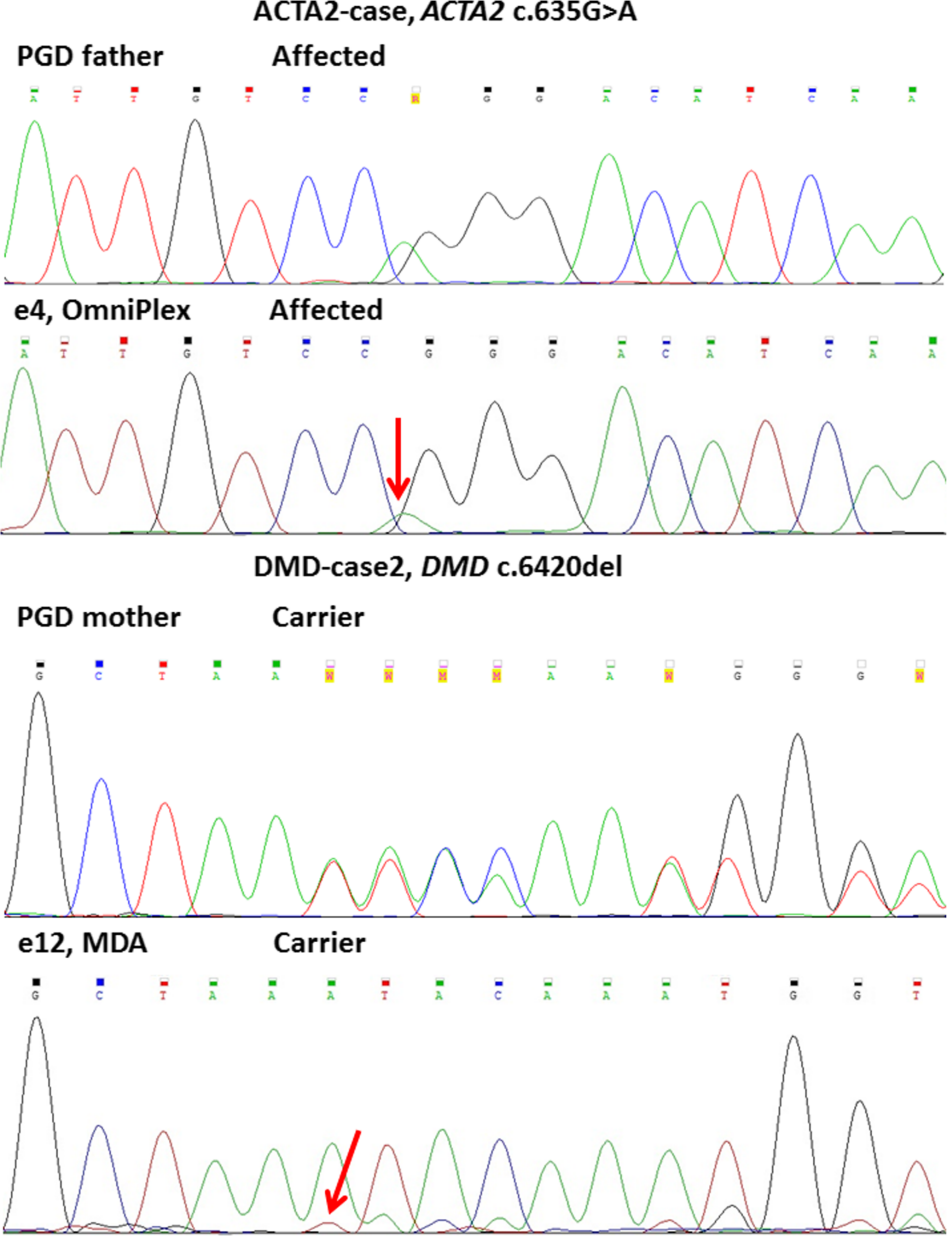
Sanger sequencing profiles of different WGA technologies. ACTA2-case and DMD-case 2 are shown. PGD father (first upper panel) and PGD mother (third panel) represent locus controls performed on gDNA. Haplotype analysis of both given embryos corresponds to heterozygous genotype (confirmed by STR haplotyping). Red arrows mark partial loss (partial allelic dropout, ADO) of disease-causing allele in analysed embryos, which are only detectable as weak background profiles similar to noise

Our results show that both WGA methodologies result in partial ADO when Sanger sequencing is performed (Fig. 2). Poor amplification of disease-causative alleles can be distinguishable as a low-level electropherogram in otherwise clear profiles. One TPP1-case sample resulted in complete disease causative allele ADO even despite a hemi-nested amplification approach. ADO rates (partial ADO, since only one complete ADO was detected) for Sanger sequencing are higher in the case of OmniPlex compared to MDA (10 and 21.4% accordingly).

**Fig. 3:**
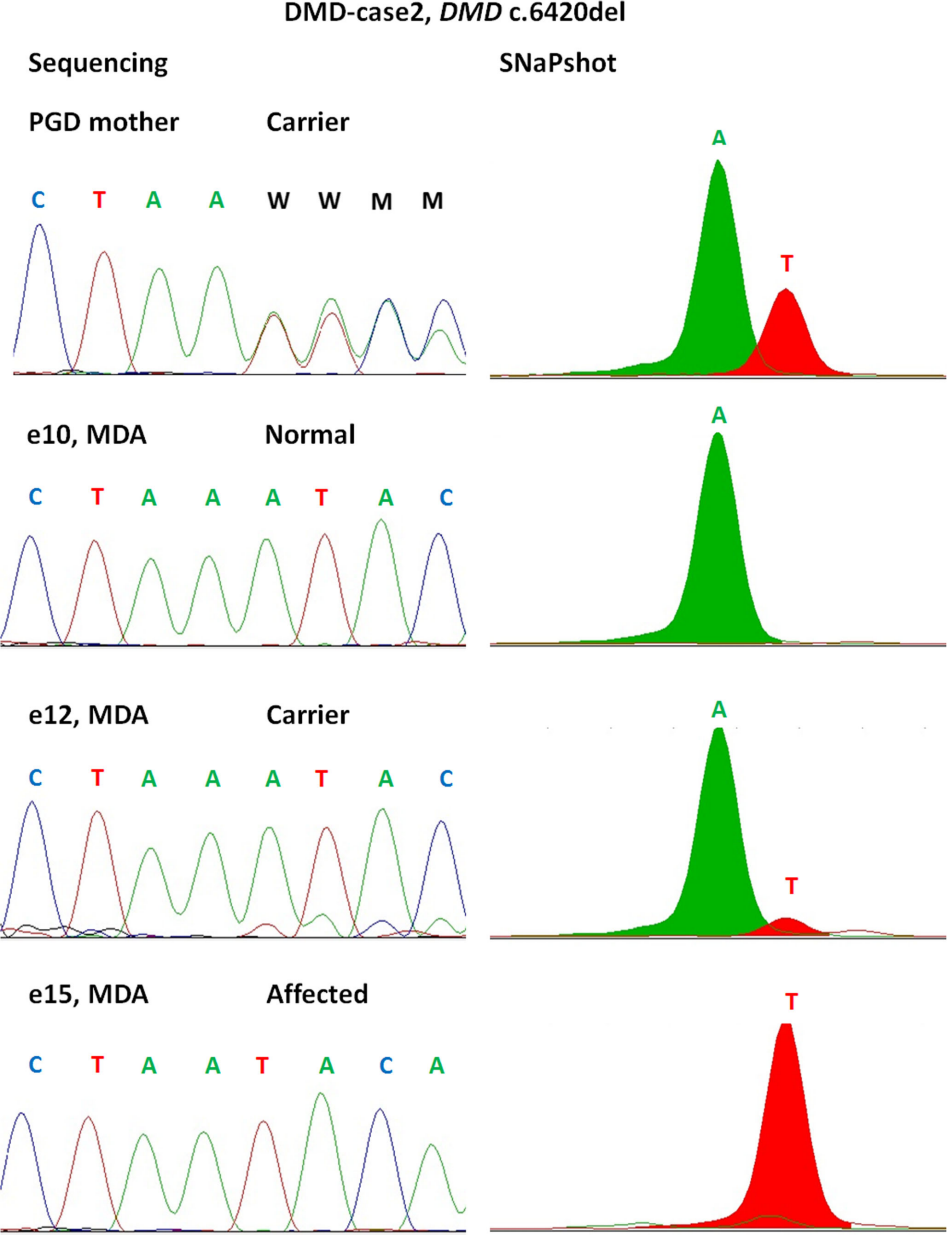
Comparison of STR sizing (A) and SNaPshot (B). Results shown for DMD-case 2 variant locus (*DMD* c.6420del). Whole genome amplification performed by multiple displacement amplification (MDA) technique for three embryos shown (e10, e12, e15). Profiles completely match between two technologies, and partial allelic dropout is visible on both profiles for the heterozygous embryo (e12). PGD mother (first upper panel) shows locus control performed on gDNA

We were also interested in comparing both WGA when subjected to SNaPshot genotyping technology (Fig. 3, Supplementary Fig. 8). The MDA product resulted in comparable results to haplotyping and Sanger sequencing, and all the genotypes matched, with an ADO rate for MDA SNaPshot 5.5%, whereas the OmniPlex product repeatedly did not produce any reliable profiles or failed to amplify completely (not shown) in more than 60% of samples.

**Fig. 4:**
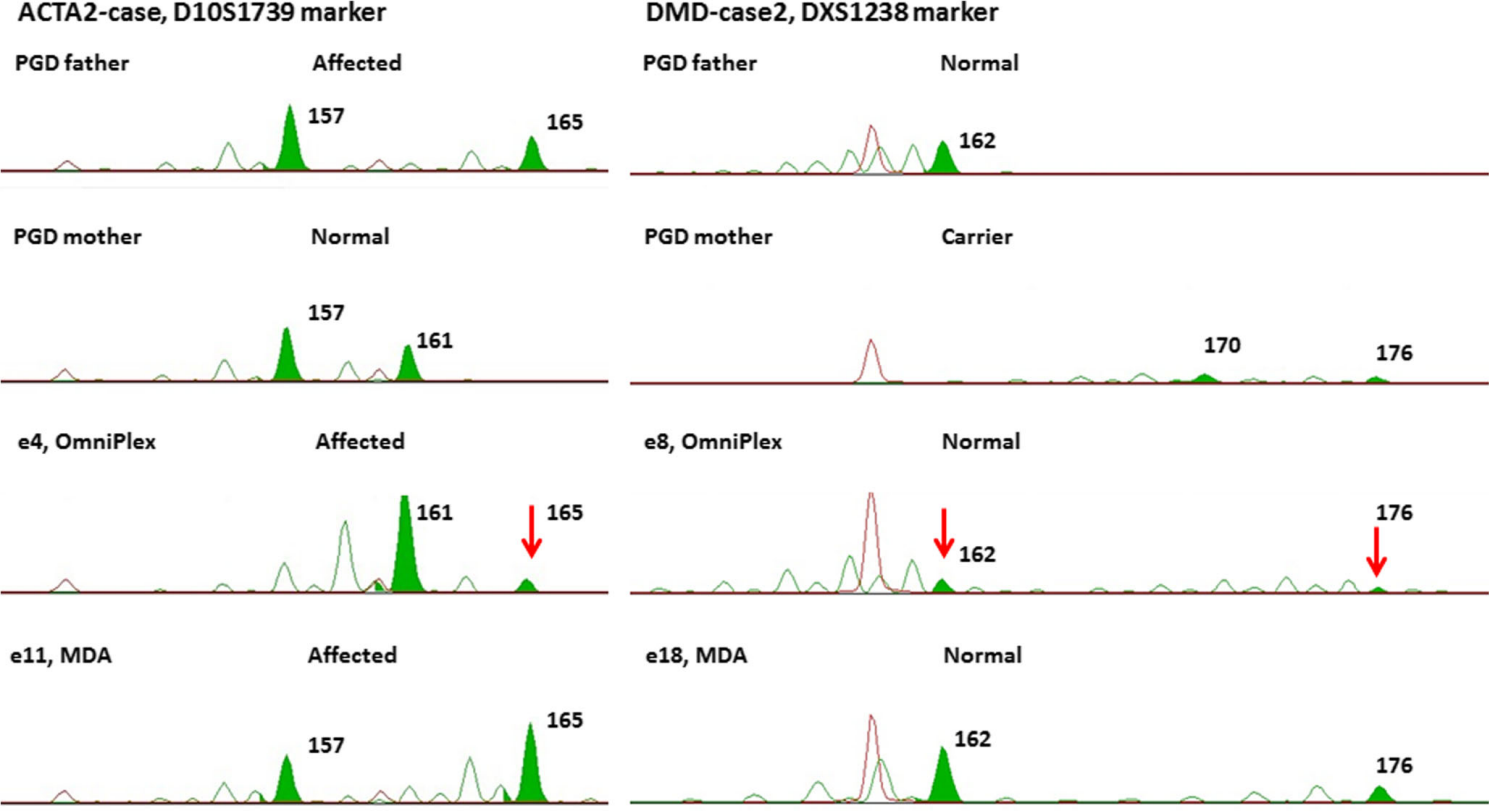
STR haplotyping analysis. Two STR marker analysis shown for ACTA2-case (left panel) and DMD-case 2 (right panel). Green peaks indicate true paternal or maternal alleles. It is visible that in the case of the OmniPlex amplifier (e8), higher peaks are of artificial nature arising due to polymerase slippage during WGA and STR amplification reactions with subsequent preferential amplification of wrong-size allele

**Fig. 5:**
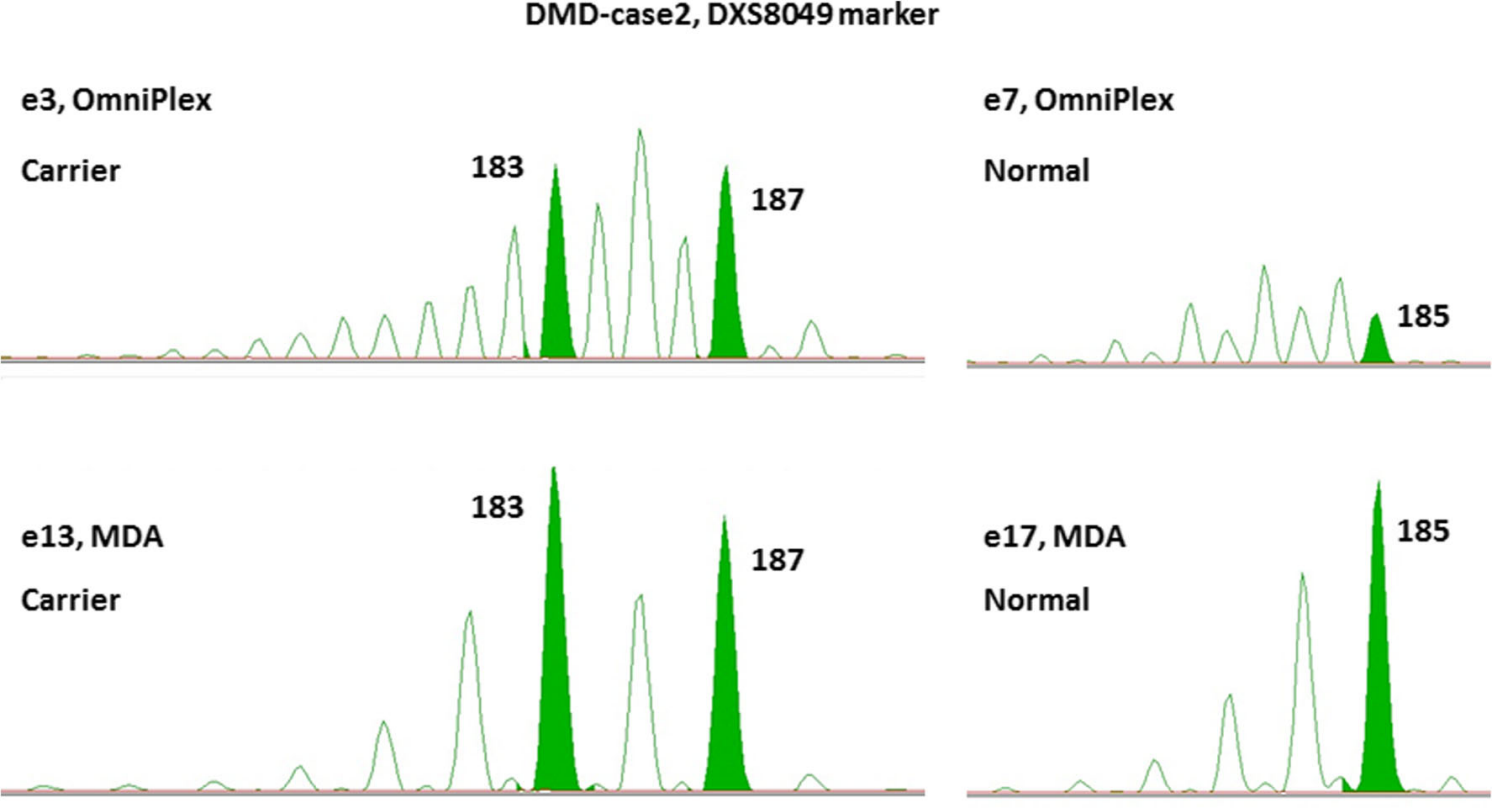
Comparison of two WGA techniques in downstream DXS8049 STR marker sizing. During STR amplification, the OmniPlex product produces much more stutter peaks (e3, e7) compared to the MDA product (e13, e17). Green peaks indicate true inherited alleles

Due to the nature of two WGA types, they arise in different downstream STR amplification product sizing patterns performed on capillary electrophoresis. Much prominent false peaks arise due to polymerase slippage during OmniPlex amplification and subsequent preferential amplification of particular PCR products, making it possible to distinguish true alleles from the false ones only by comparing them to parental genomic DNA samples run in parallel (Figs. 4 and 5).

**Fig. 6:**
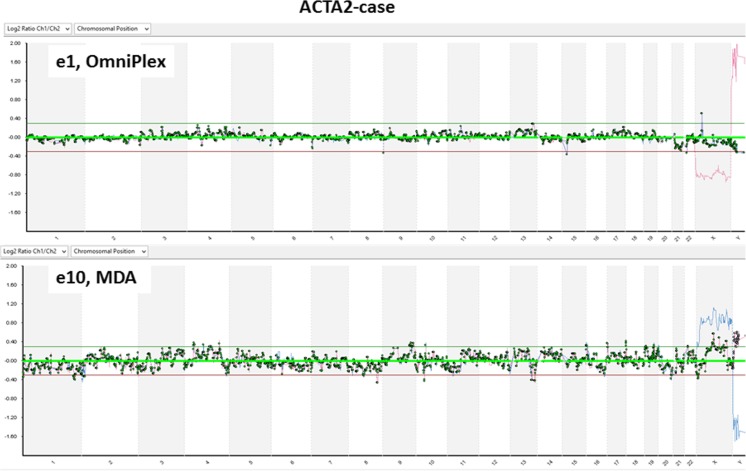
Comparison of two WGA techniques in downstream aCGH analysis

Rubicon Genomics technology has been proven to perform well in single-cell aCGH amplifications resulting in clear flat profiles, which is true in our study as well. Whereas the MDA aCGH profile results in increased noise compared to OmniPlex WGA (Fig. 6); therefore, chromosome microarray analysis in such case is possible only for the whole chromosomes, but not the partial copy number variations.

## Discussion

It is widely recognized that development of preimplantation genetic testing protocols is time consuming, costly and laborious, because of a wide spectrum of technical complications and biology-driven obstacles [3, 36]. It is essential to remember that the interpretation of results may influence not only particular family wealth but also in the long term even the well-being of the whole society, since PGT is a potential tool to cease out at least some genetic conditions.

Performance and outcome requirements for our approach were subjected to the following measures: possibility to combine several technologies in order to distinguish a normal embryo from a carrier and an affected one, to distinguish possible contamination and loci/allelic dropout events and to perform embryo chromosome screening. It was relevant to get a conclusion on all embryos subjected to biopsy and avoid any additional embryo manipulations like repeated thawing and rebiopsy. Such a wide spectrum of requirements was set carefully taking into consideration all the previous historical obstacles of PGT. We aimed first to meet the highest safety standards and secondly to prioritize a purpose of achieving the desired pregnancy in a personalized and customized manner saving patient time and expenses, which was shown through a comprehensive comparison of two different WGA techniques.

In general, obtaining micrograms of DNA through WGA of day-5 embryo biopsies allowed us to perform embryo haplotype analysis, aneuploidy screening by aCGH and direct mutation testing through SNaPshot, Sanger sequencing or fragment size analysis. As shown before, direct variant locus testing boldly complements the indirect one [18, 21], since crossover events cannot be completely ruled out and ignored [11] especially in the case of ADO, which was also true in our cohort. We designed hemi-nested primers for all assessed loci following PGDIS guidelines for good practice in PGD [16, 24]. We conclude that having as much as possible a number of semi-informative and/or informative linked markers within reasonable distance upstream and downstream from a gene is the best way to minimize the risk of misdiagnosis or no conclusive diagnosis for a particular embryo.

To our knowledge, our work is the first attempt in evaluating Picoplex and MDA amplifier performance across different downstream applications in frame of embryo preimplantation genetic testing. Provided figures give insight in understanding the applicability of both WGA methodologies to different molecular techniques and assist in choosing one when customizing PGT depending on the mutation type and technical equipment of the laboratory.

Currently, single-/few-cell WGA might be done with a wide array of amplification strategies [2]. We conclude that methodology choice depends on multiple factors like desired downstream application techniques as well as embryo amount. STR analysis efficacy including possible ADO event detection depends mostly on particular genomic region nucleotide composition and can be improved through PCR reaction condition optimization. The MDA WGA product compared to OmniPlex produces more heavy DNA strings, thus exhibiting properties closer to genomic DNA, and therefore, electropherograms are much clearer. Our results are consistent with other group findings that the per base error rate for MDA is at least two times lower compared to PCR-based approaches as shown for Sanger sequencing and SNaPshot applications. In general, MDA shows better genome recovery sensitivity as also concluded before [17] while allowing for a more convenient genotyping. However, MDA results in significant amplification bias [7], which contributed to the observed high aCGH noise levels. For full-fledged analysis, we recommend usage of both WGA techniques dividing the embryo cohort if the embryo amount is big enough. If the number of (semi-)informative markers is low, it is favourable to use the MDA technique since this will result in more robust SGD locus analysis. If STR marker informativenes is high enough, ADO will not drastically affect the result when detecting possible crossover events, and one might consider using OmniPlex since it gives more reliable aCGH profiles.

It is known that embryo aneuploidy and implantation potential are highly correlated with biopsy stage. Cleavage-stage embryo blastomere biopsy still represents the most commonly used method in Europe, although this approach has been shown to have a negative impact on embryo viability and implantation rates [5, 29]. Therefore, day 5 biopsy is highly favourable. In our study, trophectoderm biopsy performance was additionally complemented by usage of a time-lapse embryo imaging system, which not only aims at biopsy timing, but also can give a clue for the best choice of developing embryo for transfer through assessment of embryo rating by a time-lapse system algorithm when multiple embryos are SGD free and euploid.

Our experience with preimplantation testing began with a lot of goals and aims that were expected from a clinical and molecular point of view. We tried to set up a diagnostic algorithm that would suit every case and be foolproof. It became apparent already with our first cases that the approach should be more patient tailored than universal and more based on close communication between patients, clinical geneticists, reproductologists, embryologists and molecular geneticists than on pure data analysis. Proper genetic counselling before planning a PGT case is crucial as the patient has to be acquainted to any potential pitfalls to give a fully informed consent for testing. The final strategy of molecular testing should better be made after taking into consideration available embryo amount and morphology, type of disorder and family specifics and preferences. Although the main goal during monogenic disease preimplantation testing would always be disease-causative variant-free embryo selection, we found it expedient to use aneuploidy testing besides morphological embryo evaluation to determine the embryo most suitable for eSET thus increasing the chance for successful embryo implantation and development saving extra efforts and costs. The final result will always depend on a lot of different factors—even after all embryo testing is done, there is a possibility of failed implantation due to maternal age factor, endometrial receptivity problems and many more—this is why a multidisciplinary approach is a key to success for each family and thus the community altogether.

## Conclusions

Single blastocyst biopsy whole genome amplification ensures possibility of multifactor preimplantation genetic testing without compromising embryo viability and in general chance of achieving a healthy pregnancy. A semi-nested direct and indirect testing system minimizes embryo misdiagnosis risk due to allelic dropout, nonspecific amplification or contamination. 24-Chromosome aneuploidy screening when performed concurrently with single gene disorder preimplantation embryo testing provides valuable information for embryo selection excluding leading failed embryo implantation cause and notably improving single embryo transfer rates thus saving time and money leading to higher pregnancy rates. A developed protocol can be further applied to customize PGT protocols for families seeking alternatives for prenatal testing.

## Electronic supplementary material

Supplementary figure 1 (GIF 375 kb)

High Resolution (TIF 115 kb)

Supplementary figure 2 (GIF 251 kb)

High Resolution (TIF 63 kb)

Supplementary figure 3 (GIF 183 kb)

High Resolution (TIF 47 kb)

Supplementary figure 4 (GIF 283 kb)

High Resolution (TIF 67 kb)

Supplementary figure 5 (GIF 197 kb)

High Resolution (TIF 47 kb)

Supplementary figure 6 (GIF 143 kb)

High Resolution (TIF 35 kb)

Supplementary figure 7 (GIF 98 kb)

High Resolution (TIF 25 kb)

Supplementary figure 8 (GIF 329 kb)

High Resolution (TIF 113 kb)

Supplementary figure 9 (GIF 231 kb)

High Resolution (TIF 244 kb)
