# Supplementary figures and images for: Performance comparison of two whole genome amplification techniques in frame of multifactor preimplantation genetic testing
Source: J Assist Reprod Genet. 2018 Apr 23;35(8):1457–72. doi: 10.1007/s10815-018-1187-4 (PMC6086788; doi:10.1007/s10815-018-1187-4)

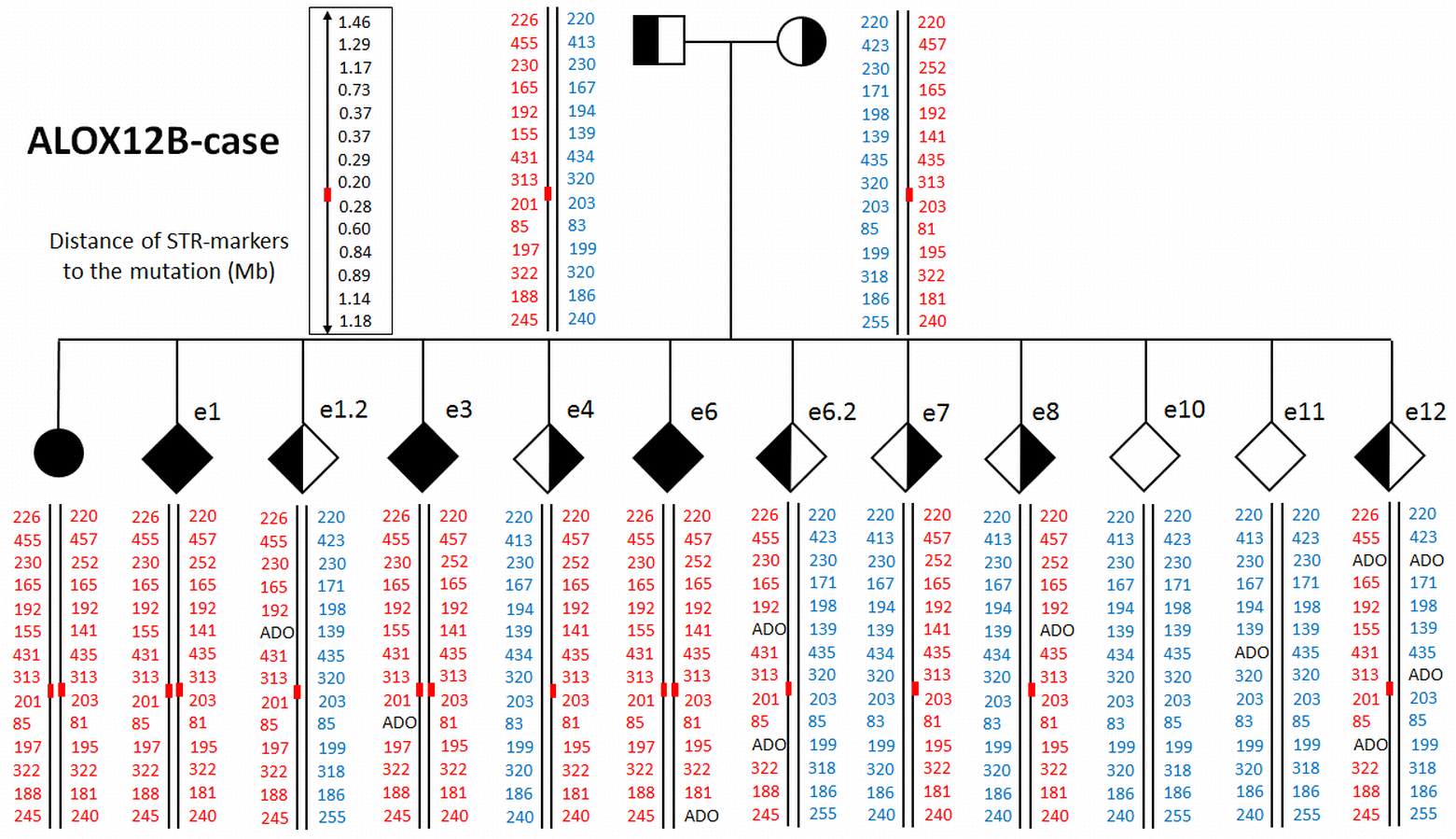

Supplement: Supplementary file 1 — (GIF 375 kb) [file 10815_2018_1187_Fig7_ESM.gif]

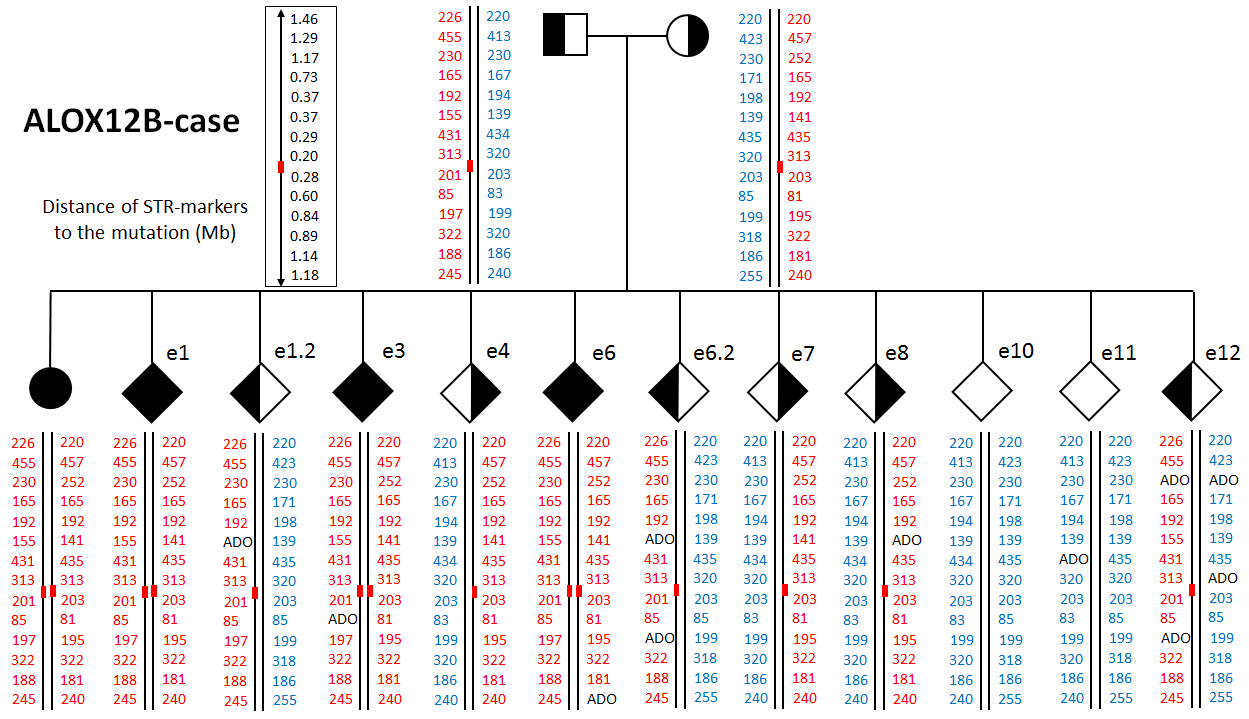

Supplement: Supplementary file 2 — High Resolution (TIF 115 kb) [file 10815_2018_1187_MOESM1_ESM.tif]

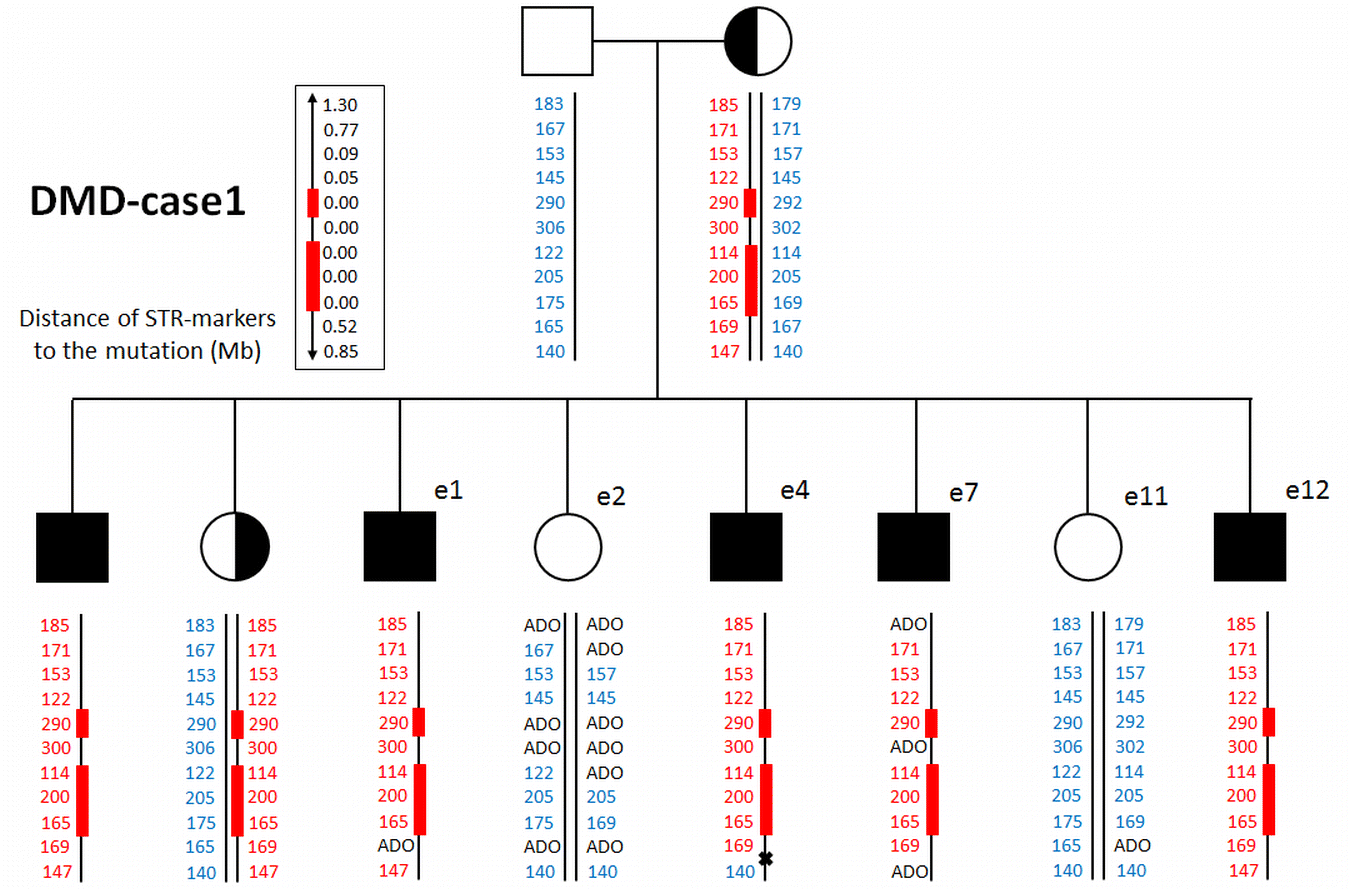

Supplement: Supplementary file 3 — (GIF 251 kb) [file 10815_2018_1187_Fig8_ESM.gif]

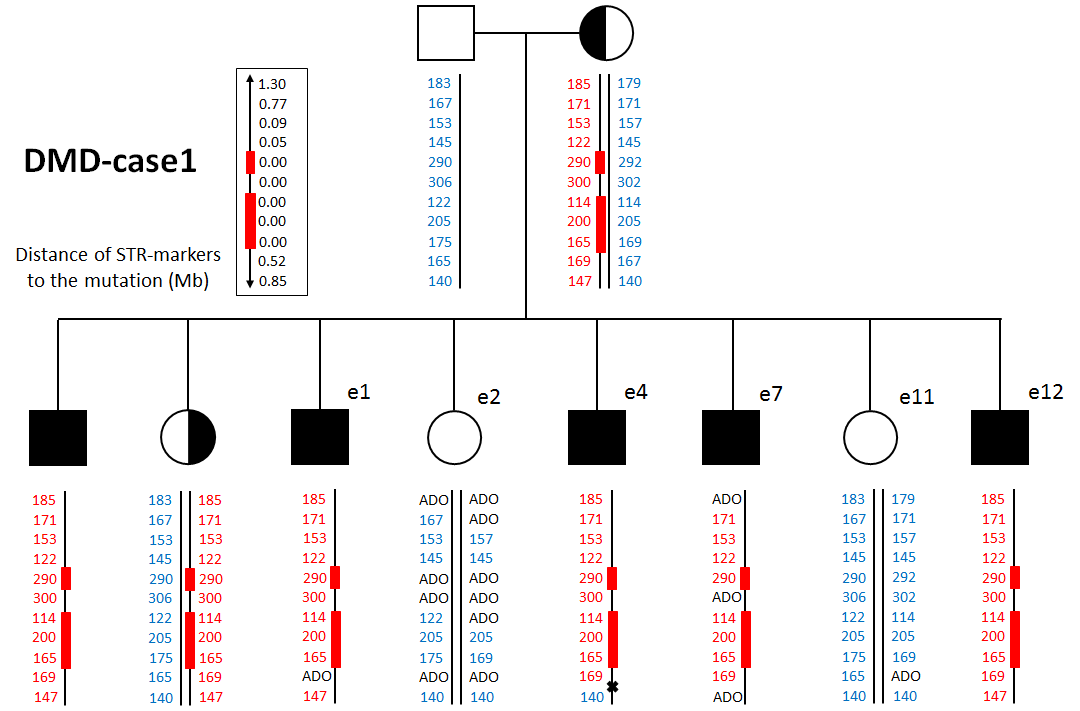

Supplement: Supplementary file 4 — High Resolution (TIF 63 kb) [file 10815_2018_1187_MOESM2_ESM.tif]

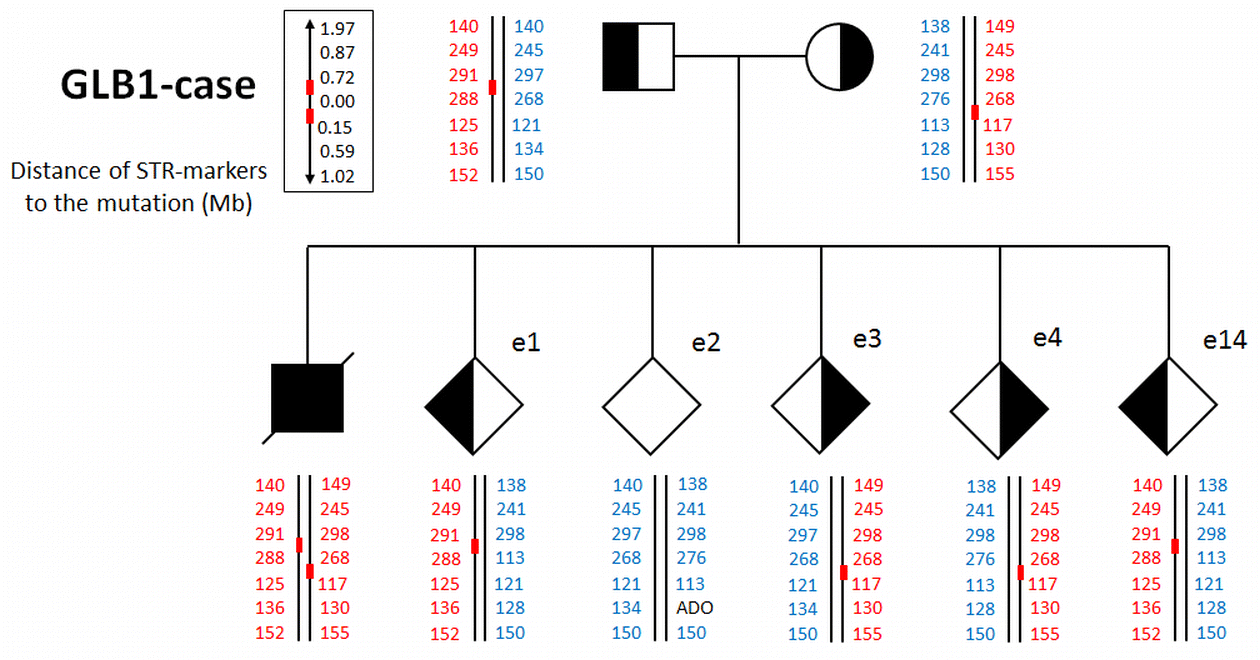

Supplement: Supplementary file 5 — (GIF 183 kb) [file 10815_2018_1187_Fig9_ESM.gif]

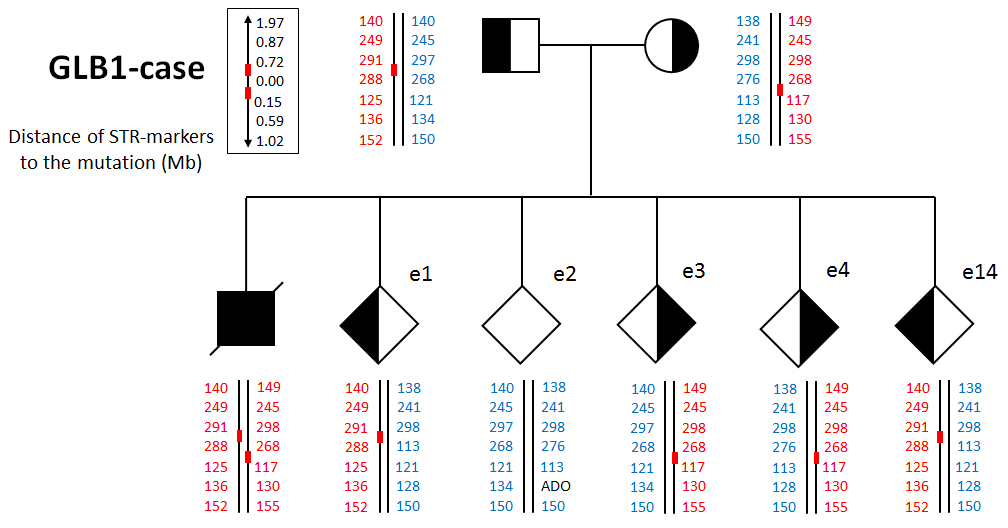

Supplement: Supplementary file 6 — High Resolution (TIF 47 kb) [file 10815_2018_1187_MOESM3_ESM.tif]

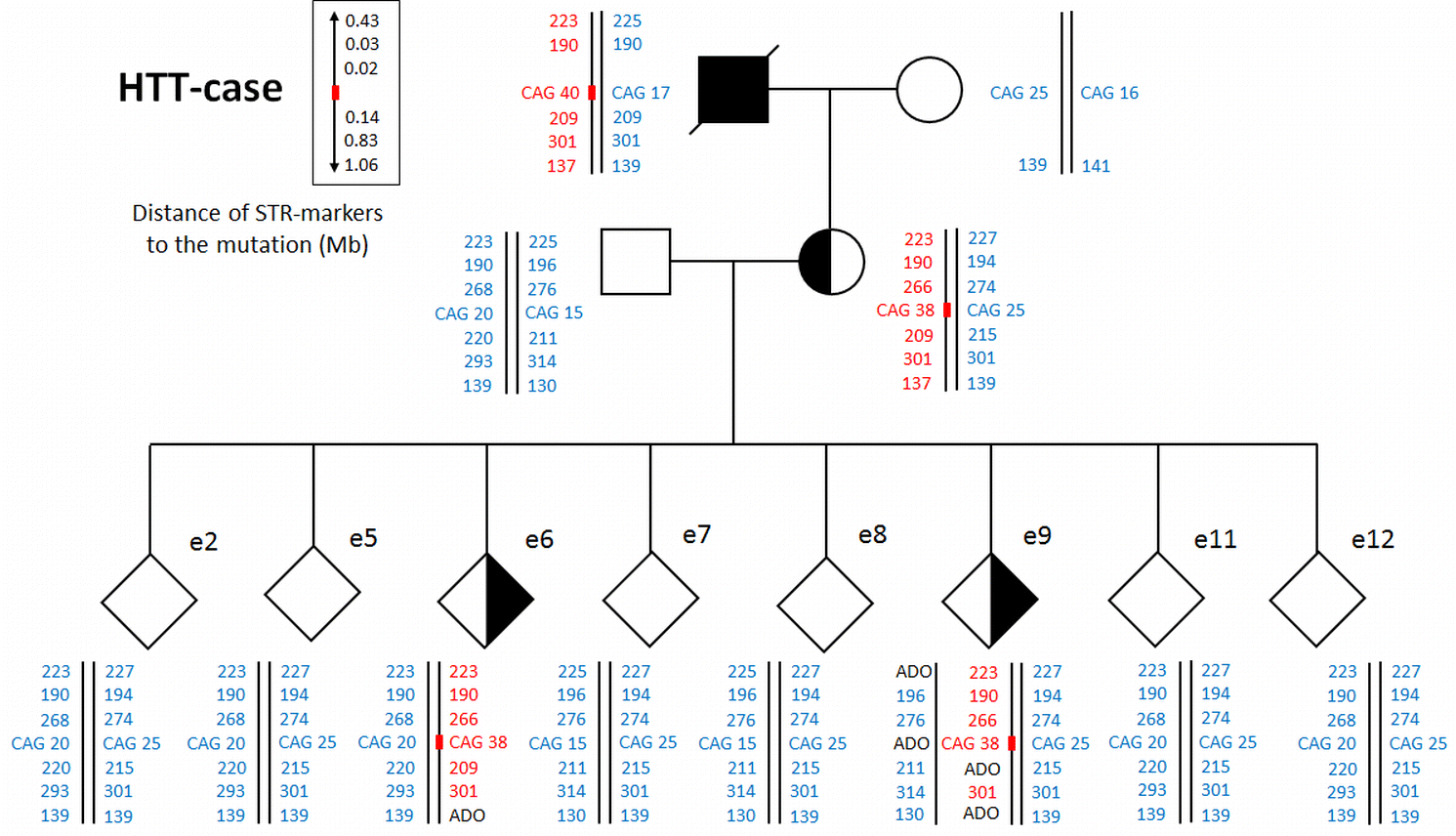

Supplement: Supplementary file 7 — (GIF 283 kb) [file 10815_2018_1187_Fig10_ESM.gif]

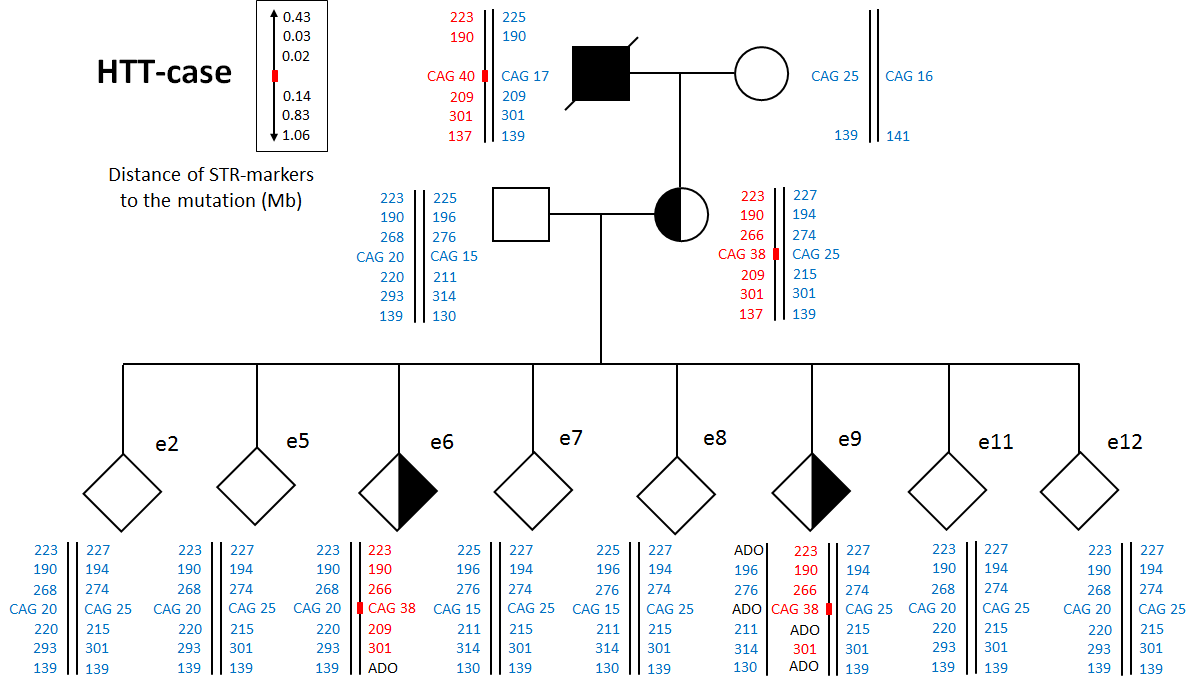

Supplement: Supplementary file 8 — High Resolution (TIF 67 kb) [file 10815_2018_1187_MOESM4_ESM.tif]

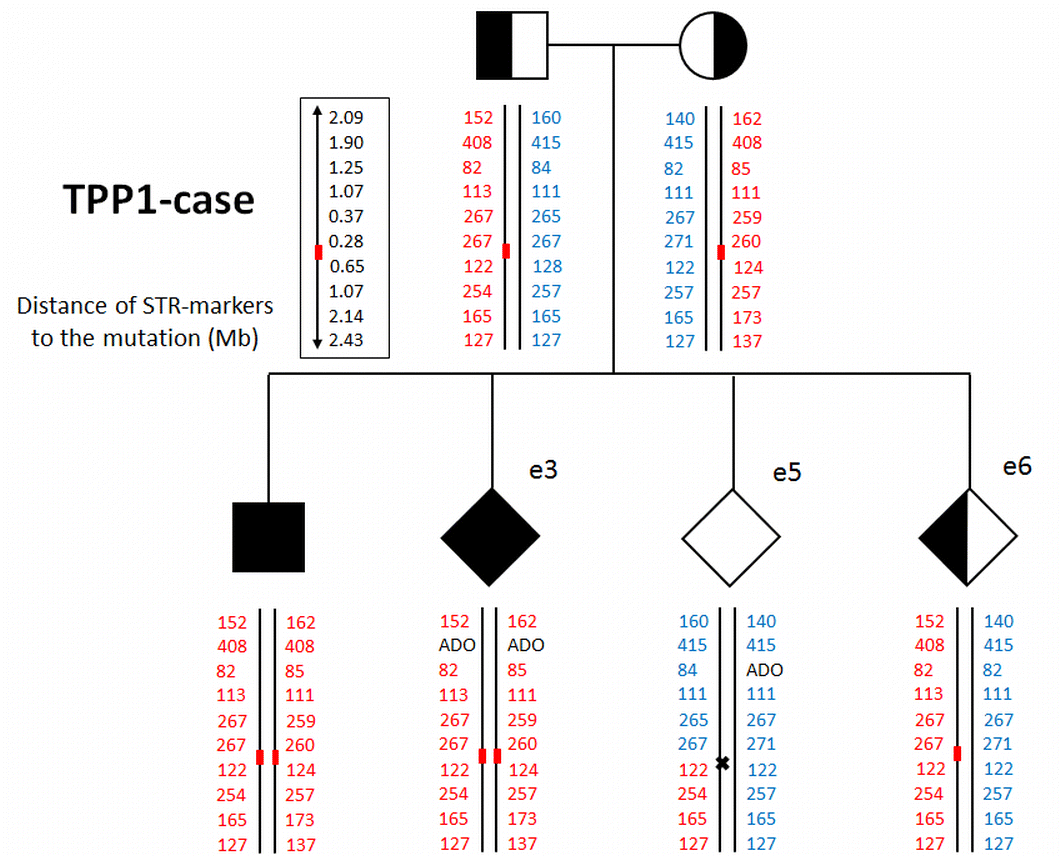

Supplement: Supplementary file 9 — (GIF 197 kb) [file 10815_2018_1187_Fig11_ESM.gif]

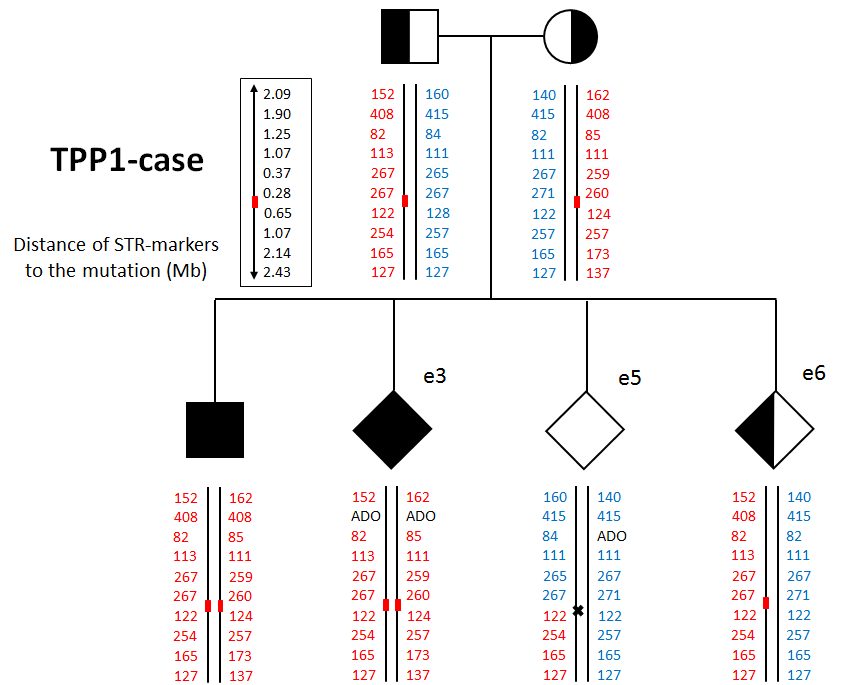

Supplement: Supplementary file 10 — High Resolution (TIF 47 kb) [file 10815_2018_1187_MOESM5_ESM.tif]

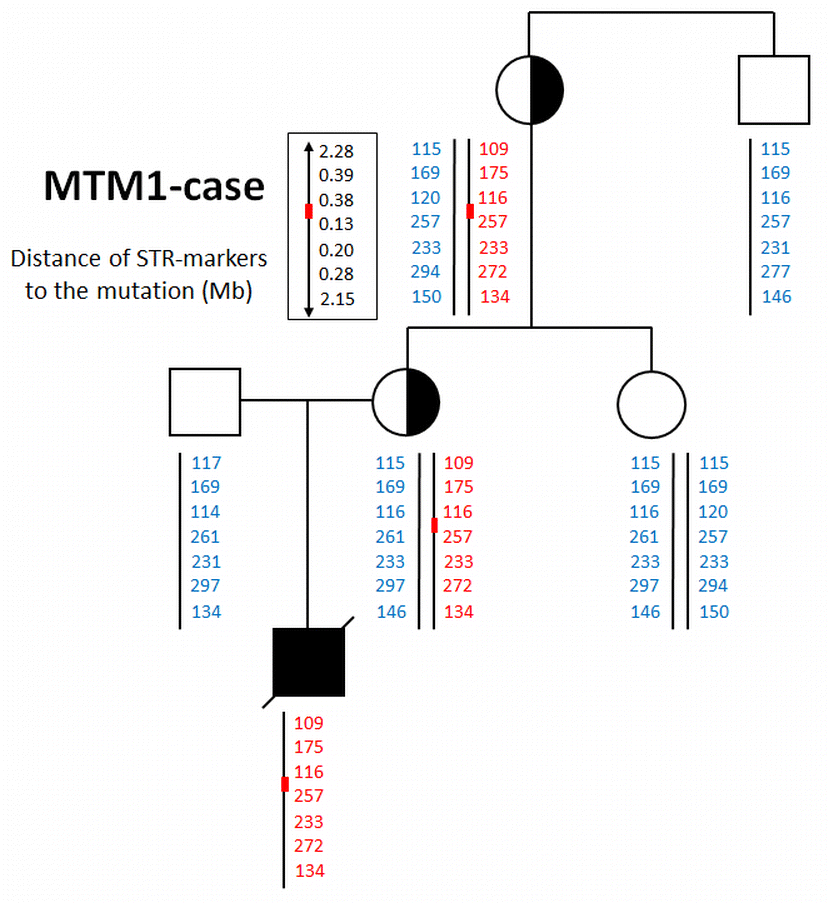

Supplement: Supplementary file 11 — (GIF 143 kb) [file 10815_2018_1187_Fig12_ESM.gif]

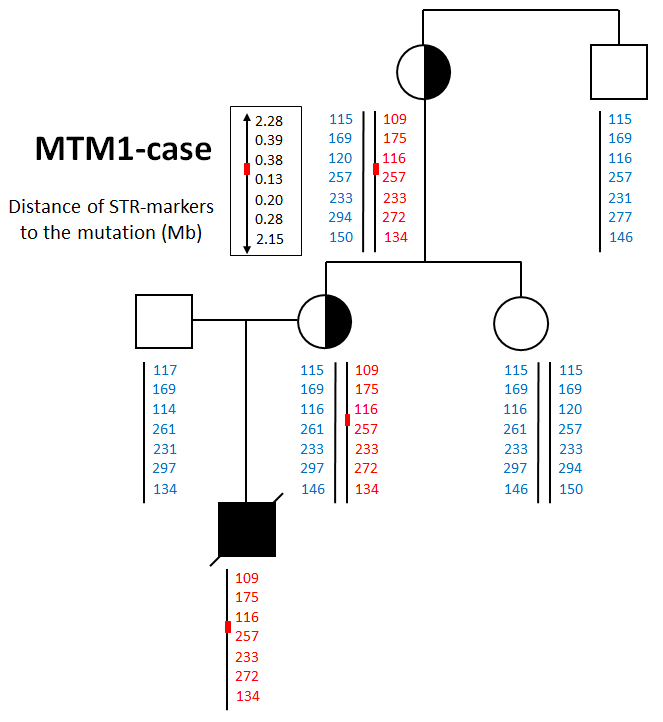

Supplement: Supplementary file 12 — High Resolution (TIF 35 kb) [file 10815_2018_1187_MOESM6_ESM.tif]

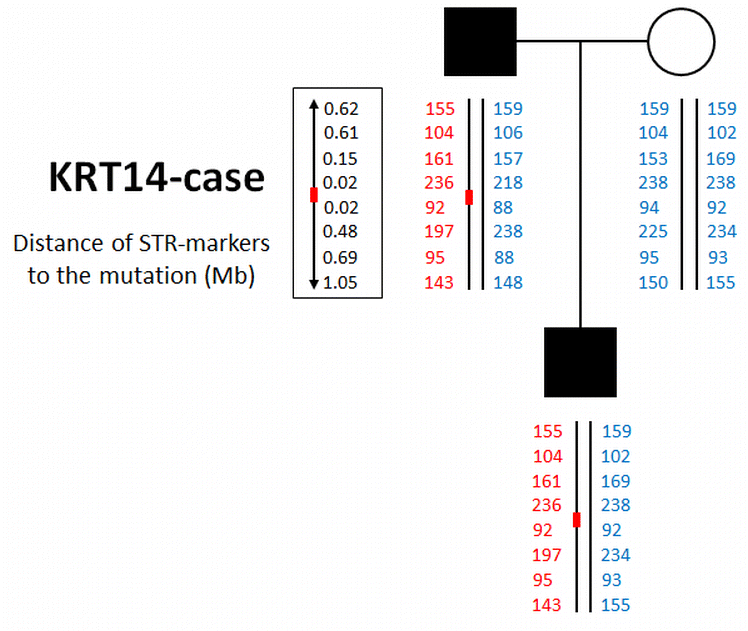

Supplement: Supplementary file 13 — (GIF 98 kb) [file 10815_2018_1187_Fig13_ESM.gif]

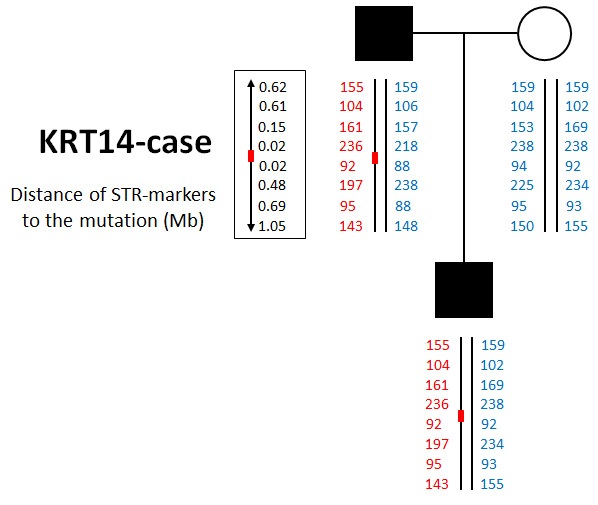

Supplement: Supplementary file 14 — High Resolution (TIF 25 kb) [file 10815_2018_1187_MOESM7_ESM.tif]

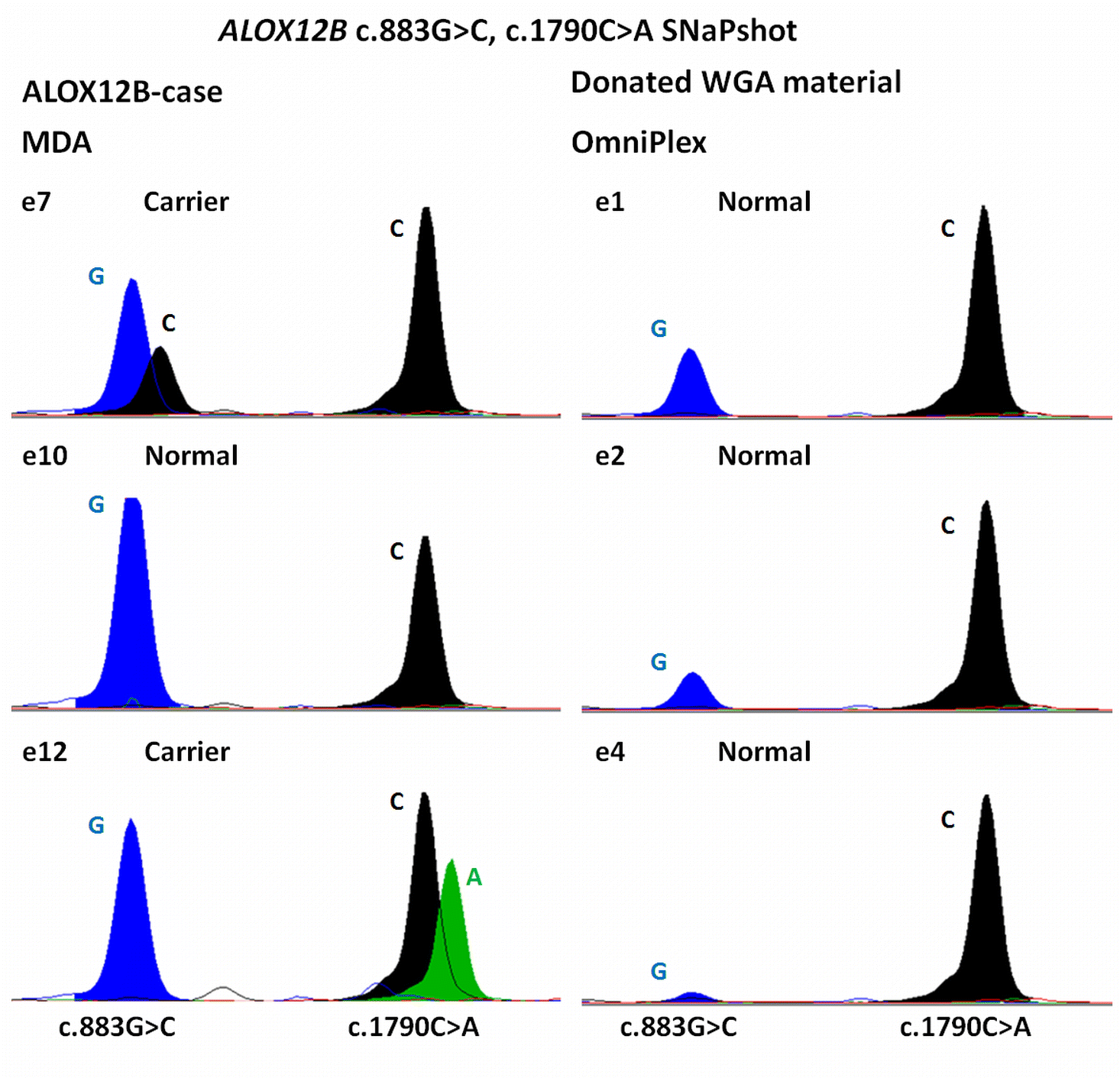

Supplement: Supplementary file 15 — (GIF 329 kb) [file 10815_2018_1187_Fig14_ESM.gif]

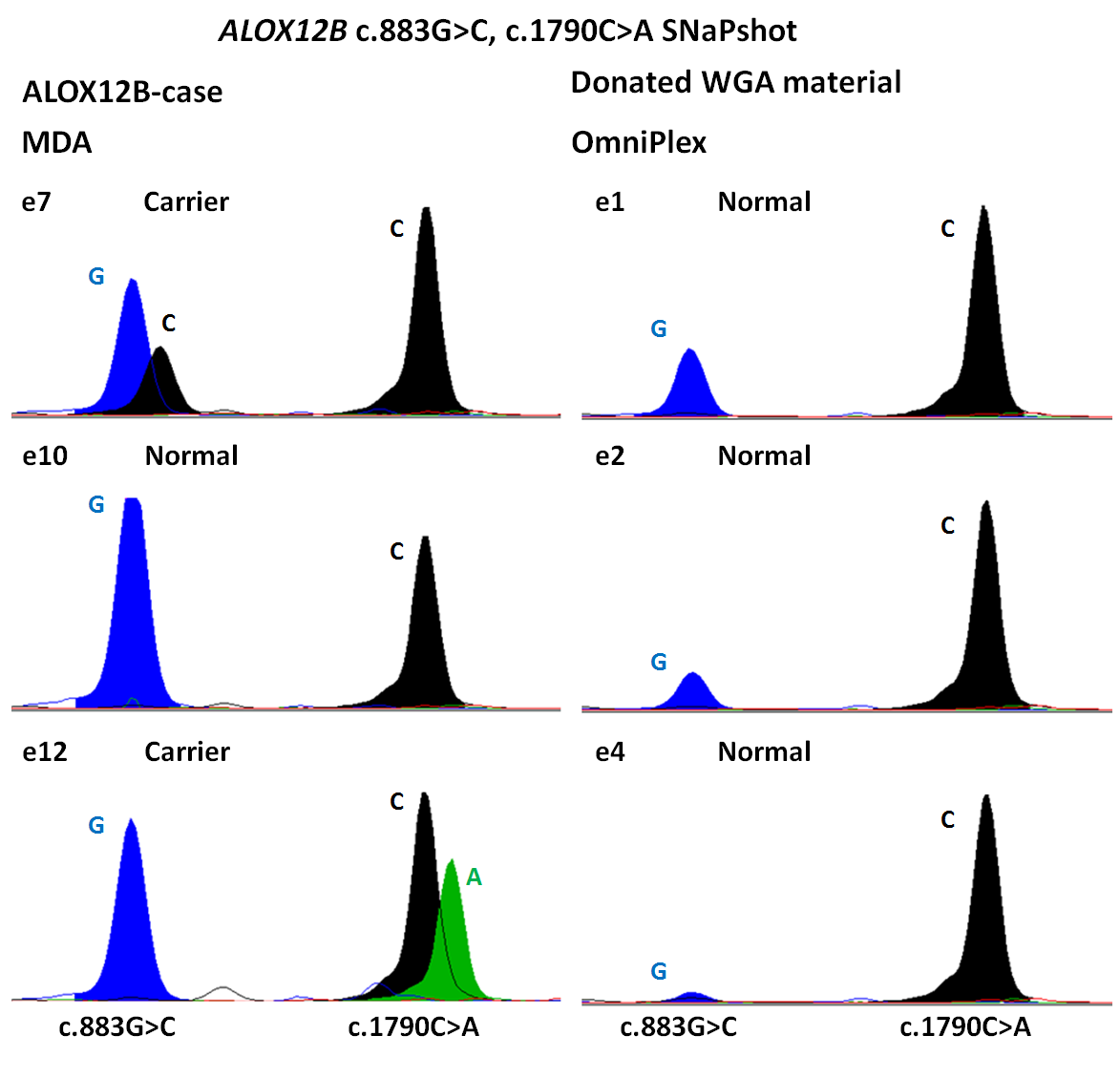

Supplement: Supplementary file 16 — High Resolution (TIF 113 kb) [file 10815_2018_1187_MOESM8_ESM.tif]

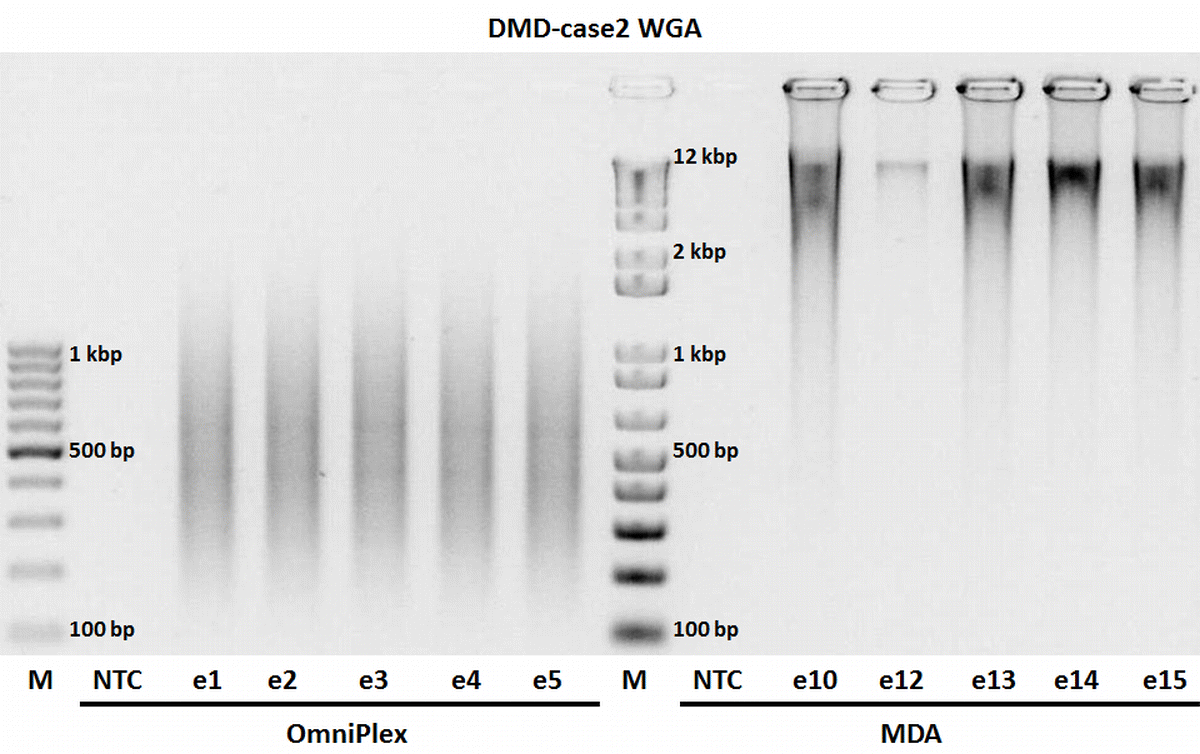

Supplement: Supplementary file 17 — (GIF 231 kb) [file 10815_2018_1187_Fig15_ESM.gif]

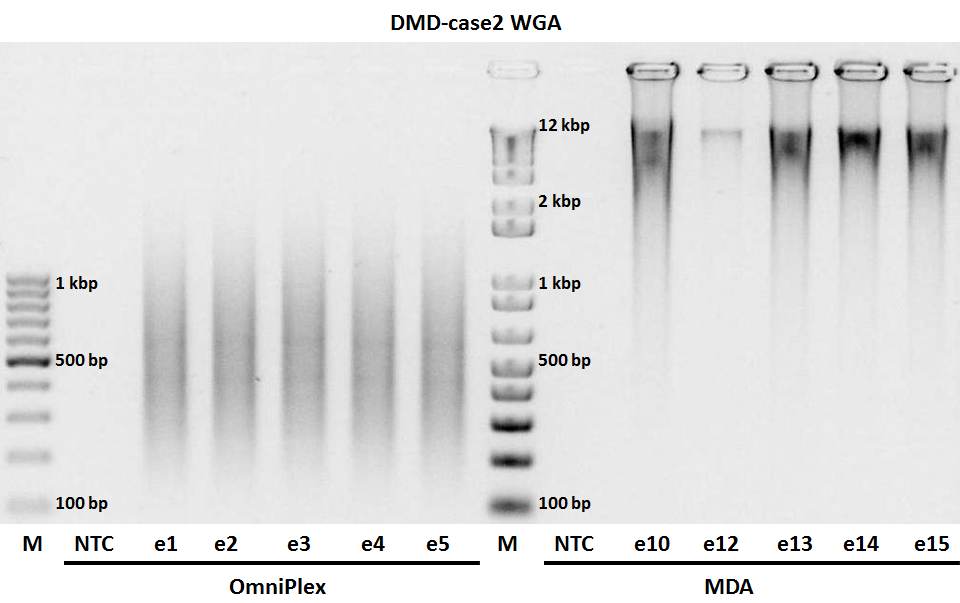

Supplement: Supplementary file 18 — High Resolution (TIF 244 kb) [file 10815_2018_1187_MOESM9_ESM.tif]
